# SSGraphCPI: A Novel Model for Predicting Compound-Protein Interactions Based on Deep Learning

**DOI:** 10.3390/ijms23073780

**Published:** 2022-03-29

**Authors:** Xun Wang, Jiali Liu, Chaogang Zhang, Shudong Wang

**Affiliations:** 1 College of Computer Science and Technology, China University of Petroleum, Qingdao 266555, China; wangsyun@upc.edu.cn (X.W.); s19070017@s.upc.edu.cn (J.L.); s20070030@s.upc.edu.cn (C.Z.); 2 State Key Laboratory of Computer Architecture, Institute of Computing Technology, University of Chinese Academy of Sciences, Beijing 100080, China

**Keywords:** deep learning, compound-protein interactions, compound properties, protein preperties, IC50 value

## Abstract

Identifying compound-protein (drug-target, DTI) interactions (CPI) accurately is a key step in drug discovery. Including virtual screening and drug reuse, it can significantly reduce the time it takes to identify drug candidates and provide patients with timely and effective treatment. Recently, more and more researchers have developed CPI’s deep learning model, including feature representation of a 2D molecular graph of a compound using a graph convolutional neural network, but this method loses much important information about the compound. In this paper, we propose a novel three-channel deep learning framework, named SSGraphCPI, for CPI prediction, which is composed of recurrent neural networks with an attentional mechanism and graph convolutional neural network. In our model, the characteristics of compounds are extracted from 1D SMILES string and 2D molecular graph. Using both the 1D SMILES string sequence and the 2D molecular graph can provide both sequential and structural features for CPI predictions. Additionally, we select the 1D CNN module to learn the hidden data patterns in the sequence to mine deeper information. Our model is much more suitable for collecting more effective information of compounds. Experimental results show that our method achieves significant performances with RMSE (Root Mean Square Error) = 2.24 and $R^{2}$ (degree of linear fitting of the model) = 0.039 on the GPCR (G Protein-Coupled Receptors) dataset, and with RMSE = 2.64 and $R^{2}$ = 0.018 on the GPCR dataset RMSE, which preforms better than some classical deep learning models, including RNN/GCNN-CNN, GCNNet and GATNet.

## 1. Introduction

The effective identification of compound-protein interactions (CPIs) plays an important role in drug design and phage biology [1]. The discovery of unknown CPIs, namely drug repositioning or drug screening [2,3], contributes to the discovery of new uses and potential side effects of drugs, which not only provides valuable insights for the understanding of drug action and off-target adverse events, but also greatly reduces the time-consuming and laborious process of traditional clinical trial methods [4]. Compounds can be represented by a Simplified Molecular Input Line Entry Specification (SMILES) string sequence [5] and 2D molecular graph with atoms as nodes and chemical bonds as edges; proteins are represented by sequences of amino acids. CPI indicates that the compounds have positive or negative effects on the functions performed by proteins, thus affecting the development of diseases [6].

In order to predict the potential CPI, many researchers have proposed a number of methods. The traditional structure-based and ligand-based virtual screening methods, although having achieved great success, are not applicable when the 3D structure of proteins is unknown or there are too few known ligand datasets. For this reason, Bredel and Jacoby introduced a new idea called chemical genomics to predict the compound-protein interaction without considering the 3D structure of the protein [7]. From the perspective of chemical genomics, the researchers then developed a prediction method based on machine learning, which considered the chemical space, genomic space and their interactions within a unified framework. The chemical space refers to the set of all possible molecules, and the genomic space refers to the set of collective characterization, quantitative research and comparative research of all genes of organisms. For example, Jacob and Vert [8] applied the support vector machine with two nuclei and used the finite element analysis based on tensor product between chemical substructures and protein families. Yamanishi et al. [9] used a bipartite graph learning method to map compound proteins to a common eigenvector space. Bleakley and Yamanishi [10] proposed a two-part local model (BLM) using similarity measures between chemical structures and protein sequences.

Most traditional prediction methods use only simple characterization of labeled data (such as known protein structure information and available CPI) to assess the similarity between the compound and the protein and infer unknown CPIs. For example, the similarity kernel function [11] and the graphics-based SIMCOMP [12] method are used to compare different drugs and compounds, which are used to describe the drug-protein interaction spectrum. The normalized Smith Waterman score [9] was used to assess the similarity between targets (proteins).

In the field of machine learning, representation learning (RL) and deep learning (DL) are two popular methods for effectively extracting features and solving scalability problems in large-scale data analysis. RL aims to automatically learn data representation (features) from original data collected from reference and open platform, which can be more effectively utilized by downstream machine learning models to improve learning performance [13,14]. DL is a data-driven technique that has proven to be one of the best models for predicting drug target binding affinity. DeepDTA [15] uses convolutional neural network (CNNs) to extract the low-dimensional real value features of compounds, which uses a vector having eight elements to represent the features of the proteins. Three convolution layers were used for feature extraction of compounds and proteins, and finally concatenates the two feature vectors to calculate the final output through the fully connected layer. WideDTA [16] follows a similar line of thought, and it also takes advantage of two additional features, ligand maximum common structure (LMCS) and protein domains and motifs (PDM), to improve the model performance. The LMCS is obtained after the pair comparison of 2k molecules [17]. PDM refers to the motifs and profiles of each protein obtained from the PROSITE database. Multiple sequence alignment of protein sequences reveals that specific regions within the protein sequence are more conserved than others, and these regions are usually important for folding, binding, catalytic activity or thermodynamics. These subsequences are called either motifs or profiles. A motif is a short sequence of amino acids (usually 10–30 aa), while profiles provide a quantitative measure of sequences based on the amino acids they contain. GraphDTA [18] uses neural network graphs [19] for graph convolutional neural network [20] (GCN) instead of learning representative compounds of CNN. In addition, the feature vectors of compounds and proteins in DeepAffinity were extracted using recurrent neural networks (RNNs), where protein feature vectors were encoded by protein structure attribute sequence (SPS) [21]. The main advantage of deep learning is that through nonlinear transformation in each layer [22], they can better represent the original data and, thus, facilitate the learning of hidden patterns in the data. DL are now being focused on many other fields, including bioinformatics such as genomics [23] and quantitative structure-activity relationships in drug discovery [24].

In this paper, a new deep learning framework is developed which combines the local chemical environment of the sequence and the topological structure of the molecule together to predict the compound protein interaction. Specifically, proteins are represented by structural property sequence SPS (which have lower dimensions and more information than protein Pfam domains), and compounds are represented by the SMILES string and molecular graph. After that, we propose a deep learning model SSGraphCPI that combines recurrent neural networks and graph convolutional neural networks, using unlabeled data and labeled data to predict CPI. Unlabeled data refer to a compound/protein characteristic representation and are used in the pre-training section of RNN/RNN; Labeled data refer to compound-protein interactions and are used during unity training (pretraining and unity training refer to 2.2.1). The input of RNN/RNN is SPS sequence and SMILES string, and the input of GraphCNN is 2D structure diagram. In the process of unified training, the SPS/SMILES feature expressions were input into CNN to get protein and compound feature vectors, and then compound feature vectors were combined with the vector obtained by GraphCNN to get the final compound vector. The final protein vector and compound vector were input into the full connection layer to predict CPI. The experimental results show that the deep learning model proposed in this paper has a lower root mean square (RMS) error than the previous model. Later, we refer to the pre-trained SPS/SMILES model as RNN/RNN, SMILES combined with 2D structural diagrams as RNN/GCNN and SMILES/SPS/ 2D structural diagrams as RNN/RNN/GCNN.

## 2. Results

### 2.1. Evaluation Index

*RMSE*: The calculation method of the root mean square error (*RMSE*) is shown in Formula (1):

(1) $$RMSE=\sqrt{\frac{\sum_{i=1}^{n}{\left(X_{model,i}-X_{obs,i}\right)}^{2}}{n}}\;$$

where i represents the $i_{\mathrm{th}}$ test sample, $X_{obs,i}$ and $X_{model,i}$ represent the observed value and the predicted value of the model, respectively, and n represents the total experimental data. RMSE is used to represent the absolute error. The better the model effect is, the lower RMSE the model has. 

$R^{2}$: $R^{2}$ is used to evaluate the degree of linear fitting of the model. The greater $R^{2}$ is, the better the degree of fitting is. The calculation method of $R^{2}$ is shown in Formula (2):

(2) $$R^{2}=1-\frac{SS_{residual}}{SS_{totality}}\;\;\mathrm{or}\;R^{2}=\frac{SS_{regression}}{SS_{totality}}$$

where $SS_{residual}$ represents the total sample difference between the actual value and the predicted value of the model, $SS_{regression}$ represents the sum of squares of the differences between the predicted value and the mean value and $SS_{totality}$ represents the average sum of the differences between the real value and the mean value.

### 2.2. Comparison of RMS Errors Values for Different Models

To assess the impact of our newly proposed SSGraphCPI model on compound-protein interactions, we compared RMS errors in different datasets. The smaller the RMS errors are, the better accuracy the prediction model has in describing the experimental data. In addition, if SSGraphCPI and SSGraphCPI2 achieve the best or second best values, they are shown in bold in the table. If one of the two models achieves the best value and the other does not achieve the second best value, only the best value is shown in bold. Table 1 shows that the RMS errors of our SSGraphCPI model are all lower than the most advanced model in the test set, and the SSGraphCPI2 model even reaches the minimum value. As can be seen from Table 2 and Table 3, the SSGraphCPI model reaches the minimum value on the ER and Channel dataset. As can be seen from Table 4, our model shows good performance on the GPCR dataset. As can be seen from Table 5, although the Kinase dataset achieves lower RMSE and higher R than other models, the RMSE of this dataset is significantly improved compared with other datasets, indicating that the performance of the model in data specificity needs further improvement. To sum up, the SSGraphCPI model can effectively improve the accuracy of predicting compound-protein interactions, and the SSGraphCPI2 model also shows better performance on several datasets.

### 2.3. Comparison of Loss Values of Different Models

**Figure 1:**
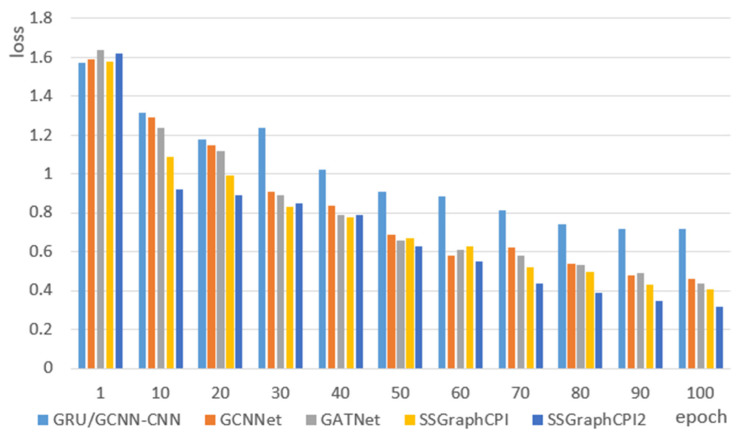
Loss value variation diagram of different models.

We compared the loss values of different models during the training process, and trained a total of 100 epochs. We took 10 epochs as one node and plotted the loss line diagrams of different models. As shown in Figure 1, the loss value of SSGraphCPI model at most epoch points was lower than that of the current most advanced model and the loss value tended to 0.41. The loss value of the SSGraphCPI2 model is much lower than that of the other models and finally tends to 0.35. It can be seen that the model proposed in this paper can effectively improve the performance of previous models and predict the compound protein interaction more accurately.

### 2.4. Model Validation

In order to verify the validity of the SSGraphCPI model, we further used the model to predict compounds with high interaction strength from the number of compounds for a specific protein. Specifically, we selected the epidermal growth factor (EGF) receptor protein, which is a heat-resistant single chain low molecular polypeptide composed of 53 amino acid residues. EGF receptor has a wide range of effects and plays an important role in the estimation of tumor prognosis and selection of treatment regimens, as well as in the treatment of gastric ulcer and liver failure.

**Figure 2:**
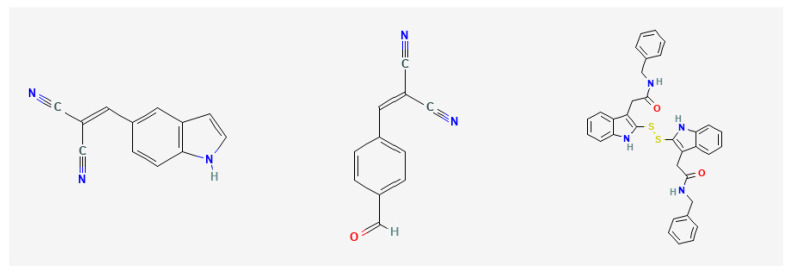
A 2D molecular diagram of the top three compounds.

**Figure 3:**
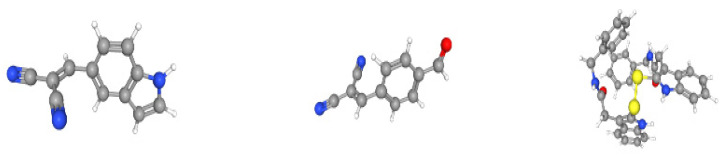
A 3D molecular diagram of the top three compounds.

**Figure 4:**
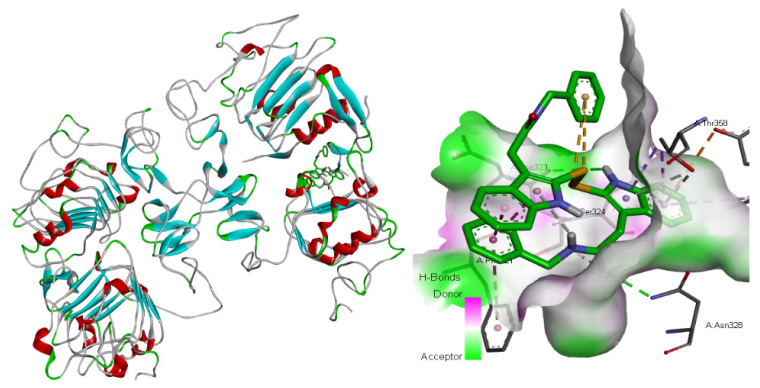
Docking diagram of C_34_H_30_N_4_O_2_S_2_ molecule and EGF receptor protein.

In total, 145 compounds with different interaction intensities with this protein were selected for prediction, and the first three predicted compounds were: C_12_H_7_N_3_, C_11_H_6_N_2_O and C_34_H_30_N_4_O_2_S_2_. The top 30 compounds known to interact with EGF proteins are shown in Table 6, with our predicted top three compounds highlighted in bold. BindingDB database showed that the first two compounds were directly related to the EGF receptor. The top three compounds we predicted ranked 2nd, 14th and 15th, respectively, among the 145 known compound-protein interaction strengths. The 2D molecular diagrams of C_12_H_7_N_3_, C_11_H_6_N_2_O and C_34_H_30_N_4_O_2_S_2_ are arranged from left to right as shown in Figure 2. The 3D structure of these three compounds is shown in Figure 3 from left to right. Furthermore, we conducted molecular docking of these three groups of compounds and proteins, and the docking results showed that the third compound reached a high docking score of −7.1 with EGF protein, and this compound ranked 15th among known interactions, as shown in Figure 4. This also shows that our model can effectively predict compound-protein interactions.

## 3. Discussion

This model is the first three-channel model that includes protein SPS sequence, SMILES string and 2D structure diagram of a compound. The input of the three channels contains physicochemical properties, sequence information and structure information, which is a very comprehensive input. Moreover, an attention mechanism is added in each channel, which can extract compound protein characteristics more effectively. 

The comparison model is different from the model in input or deep learning framework, which is more conducive to the comparison of suitable input and deep learning framework. In this paper, the random partition method is adopted in the division of training set and verification set, and further research can be made on cross verification and optimization of hyperparameters in the future. In this paper, *RMSE* and *R*^2^ were used as measurement indexes to compare the differences of different models on different datasets. It can be seen from the results that SSGraphCPI model can achieve better results on the same dataset, but there are great differences in model performance between different datasets, indicating that the sensitivity of the model on specific datasets needs to be studied.

## 4. Materials and Methods

### 4.1. Materials

#### 4.1.1. Dataset

BindingDB [25] is an open, accessible, measurable binding affinity database that focuses on the interactions between target proteins and small pharmaceutical molecules. In this paper, the Root Mean Square (RMS) error of IC50 is used to evaluate the performance of the whole model. IC50, also known as the half maximal inhibitory concentration, refers to the concentration at which a drug has a 50% inhibitory effect on protein. IC50 values are often used to measure cell resistance to drugs or cell tolerance to drugs. IC50 can be calculated in a variety of ways. We used molecular data from three public databases, namely: the compound SMILES string sequence from STITCH database [26], the protein amino acid sequence from UniRef [27] database and the compound-protein interaction data from BindingDB database. In addition, Rdkit was used to convert the SMILES sequence into a 2D molecular graph of compounds [28].

Starting with 489,280 IC50-labeled samples, we completely excluded four classes of proteins from the training set, i.e., nuclear estrogen receptors (ER; 3374 samples), ion channels (14,599 samples), receptor tyrosine kinases (34,318 samples) and G-protein-coupled receptors (GPCR; 60,238 samples), to test the generalizability of our framework. Moreover, we randomly split the rest into the training set (263,583 samples including 10% held out for validation) and the default test set (113,168 samples) without the aforementioned four classes of protein targets. The label uses the IC50 value of the compound-protein interaction.

#### 4.1.2. Feature Representation of Protein

Previously the most common protein representation for CPI classification was a 1D binary vector whose dimensions correspond to thousands [29] of Pfam domains [30] (structural units) and 1 s are assigned based on k-hot encoding [31,32]. Pfam entries include the family, domain, motif, repeat, disorder and coiled-up coil of proteins.

In this paper, we used the protein structure property sequence (SPS) to represent protein feature vectors [21]. SPS are encoded by the secondary structure, solvent accessibility, physicochemical properties (acidic/basic, polar/non-polar) and amino acid residue sequence length of proteins. The SPS sequence represents a protein, not an amino acid. SSPro is used to predict the secondary structure of each residue [33]. SSPro is a detailed study of sequence-based structural similarity, predicting secondary structure and solvent accessibility of proteins at higher fractions than other predictive tools. The SPS method identifies proteins in the same family and provides explicable protein fragments responsible for predicting affinity. Taking into account the four properties of proteins, each of which is represented by an English letter, we get 72 sets of properties, plus 4 sets of special words (such as beginning, ending, padding and not-used ones) to make up 76 sets of “alphabet”. Table 7 shows representations of these four properties. For example, the word “AEKM” implies that the secondary structure of the protein is alpha type, solvent accessibility, alkalinity and medium length. The SPS sequence representation of proteins is 100 times more compact than the amino acid sequence and overcomes the disadvantages based on the Pfam domain representation: it provides greater discrimination between proteins within the same family and provides greater interpretability of which protein segments are responsible for predicting affinity. They provide higher sequence resolution and structural detail for more challenging regression tasks. When RNN and LSTM train sequences greater than 1000 [15], the convergence problem can be avoided.

#### 4.1.3. Feature Representation of a Compound

In this study, we propose a new method to express compound eigenvectors using a combination of two methods. This method considers both the local chemical environment and the topological structure of the compound. The first representation is the compound 1D SMILES string sequence [5], which is a short ASCII string used to represent the chemical structure of a compound based on bonds and rings between atoms, such as “C1=C2C (C=C=C…)”, which is a sequence of atomic and covalent bonds. For the convenience of expression, we take both atomic and covalent bonds as symbols. Therefore, the SMILES sequence is a symbol sequence. A total of 64 symbols are used for SMILES strings in our data. Additionally, 4 more special symbols are introduced for the beginning or the end of a sequence, padding (to align sequences in the same batch) or not-used ones. Therefore, we defined a compound ‘alphabet’ of 68 ‘letters’. Compared to the baseline representation which uses k-hot encoding, canonical SMILES strings fully and uniquely determine chemical structures and are yet much more compact.

The second representation is a 2D molecular graph structure of the compound, which is converted from the SMILES string by the Rdkit tool. In this paper, we used three layers of GCNN (*R* = 3) and five different convolutional filters instead of one for atoms with different number of neighbors ($H_{1}^{1}$,…,$\;H_{R}^{5}$). For example, if an atom has n neighbors, then $H^{n}$ convolutional filter will be used for it in the CNN. The specific process is shown in Algorithm 1.
**Algorithm 1** Graph CNN. **Input:** Molecule graph *G* = (*V*, *E*), radius *R*, hidden weights $H_{1}^{1}\ldots \;H_{R}^{5}$ **Output:** A vector **r**_a_ for each atom a **Initialize:** Initialize all the **r**_a_ **For**
*L* = 1 to *R*
**do**  **For** each node a ∈ *V*
**do**   *N* = neighbors(a)   **v** ← **r**_a_ + $\underset{u\in N}{\sum }r_{u}$   **r**_a_ ← σ(**v**$H_{L}^{\left|N\right|}$)  **end for**  **end for**

### 4.2. Methods

#### 4.2.1. SSGraphCPI Model Framework

The SSGraphCPI model consists of three channels, one of which encodes the protein SPS sequence, and the remaining two channels encode the SMILES string and the 2D molecular graph of the compound, respectively. Two channels encoding a compound are integrated and combined with the channel encoding a protein to predict the compound protein interaction.

First, we use Graph Convolutional Neural Network (GCNN) to encode the 2D molecular graph of compounds. The detailed process has been described in the second part. Compound SMILES and protein SPS both used the recurrent neural network (RNN) model, seq2seq [34], which has seen much success in natural language processing and was recently applied to embedding compound SMILES strings into fingerprints [35]. A Seq2seq model is an auto-encoder that consists of two recurrent units known as the encoder and the decoder. The encoder maps an input sequence (SMILES/SPS in our case) to a fixed-dimension vector known as the thought vector. Then, the decoder maps the thought vector to the target sequence (again, SMILES/SPS here). We choose gated recurrent unit (GRU) [36] as our default seq2seq model and treat the thought vectors as the representations learned from the SMILES/SPS inputs. Our alphabets include 68 and 76 letters (including 4 special symbols such as padding in either alphabet) for compound SMILES and protein SPS strings, respectively. Based on the statistics of 95% CPIs in BindingDB, we set the maximum lengths of SMILES and SPS strings to be 100 and 152, respectively. Accordingly, we used 2 layers of GRU with both the latent dimension and the embedding layer (discrete letter to continuous vector) dimension being 128 for compounds and 256 for proteins. We used an initial learning rate of 0.5 with a decay rate of 0.99, a dropout rate of 0.2 and a batch size of 64.

By pre-training compound and protein features, nonlinear co-dependencies between protein residues or compound atoms in the sequence can be captured. “Long-term” dependence is important for compound-protein interactions because the corresponding residues or atoms can be tightly bound in 3D structures and work together to facilitate molecular interactions. The pre-training model includes embedding layer, encoder, attentional mechanism and decoder. This section uses unmarked SPS/SMILES data. The training time was 100 epochs with a learning rate of 0.001. 

In the unified model, RNN/RNN part includes the embedding layer, encoder, attention mechanism and a CNN is added after RNN/RNN, respectively. The pre-trained embedded layer and encoder parameters will be used as the initialization of the unified model, and will be co-trained with the attention mechanism, CNN and GCNN. This section uses labeled compound protein interaction data. This is equivalent to the entire model being trained with 200 epochs at a learning rate of 0.0001.

**Figure 5:**
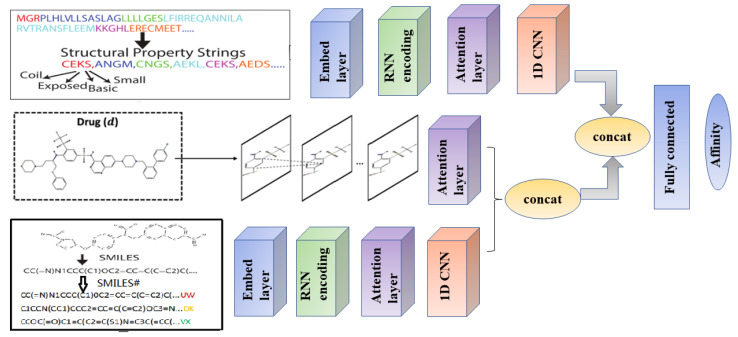
The overall flow chart of the SSGraphCPI model.

The entire SSGraphCPI pipeline is trained from end to end [37], with the pre-trained RNN/RNN serving as warm initializations, for improved performance over two-step training. The pre-trained RNN/RNN initializations prove to be very important for the non-convex training process [38]. The specific model diagram is shown in Figure 5.

#### 4.2.2. Baseline Model

We compared the SSGraphCPI model presented in this paper with the following most advanced baseline model.

RNN/GCNN-CNN [21]. In this model, the two-dimensional molecular graph of the compound and the SPS sequence of the protein were used as inputs, and the one-dimensional SMILES sequence of the compound was not considered. In this model, recurrent neural network is used to encode the SPS sequence of proteins to obtain the protein feature vectors, and the graph convolutional neural network is used to encode the molecular graph of compounds to obtain the compound feature vectors. Finally, the convolutional neural network was used to predict the protein-compound interactions.

GCNNet [39]. Similar to the method proposed in this paper, the model has three input variables, which are the protein amino acid sequence, compound 2D molecular graph and compound SMILES string. Different from this paper, GCNNet uses LSTM to encode the protein amino acid sequence and SMILES string, and finally uses the convolutional neural network to predict the compound-protein interaction. LSTM is a special RNN network, which is mainly used to solve the problem of gradient disappearance and gradient explosion in long sequence problems. In short, LSTM performs better in longer sequence problems than normal RNN networks.

GATNet [39]. This model only adds the attention mechanism to the 2D molecular graph of compounds in the GCNNet model, which then form Graph Attention Networks (GATs). The attention mechanism is applied to the graph neural network, and the contribution of each neighbor to the generation of new features at each layer of learning nodes is aggregated according to the contribution of neighbor features, so as to generate new features of nodes. GATNet has the characteristics of low computational complexity and suitable for inductive learning task.

#### 4.2.3. SSGraphCPI2 Model Framework

**Figure 6:**
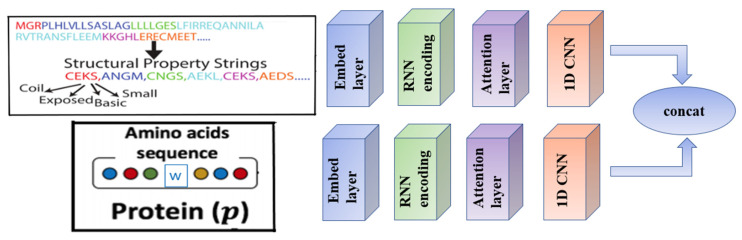
Network diagram of the protein feature vectors extraction.

We developed the SSGraphCPI2 model, which added protein amino acid sequence on the basis of the SSGraphCPI model. We also use the bidirectional GRU to extract the features of the amino acid sequence of the protein, and then use the attention model to strengthen the key sequence fragments, and enter it into the 1D convolutional neural network to obtain the feature vectors of the protein. Then, it is combined with the feature representation vector obtained from the SPS sequence of proteins to obtain the final protein characterization. It combines with the compound feature vector into the fully connected layer to predict the compound protein interaction. SSGraphCPI2 also adopts pre-training and unified training methods. SMILES and SPS channels adopt pre-training parameters in SSGraphCPI model. After the pre-training of SSGraphCPI2, the parameters of the channel with amino acid as input can be obtained. In the unified training, the RNN parameters of the above three channels were used as the initialization of the unified training, and finally all the parameters of the model were obtained. The model extracted protein information in a more comprehensive way, among which SPS sequence is about the structure and physical and chemical properties of the protein, and the amino acid sequence is about the whole context of the protein. The experimental results show that the model can effectively reduce the predicted RMS errors. The network architecture of protein feature extraction is shown in Figure 6.

## 5. Conclusions

Accurately predicting CPI is an important and challenging task in drug discovery. In this article, we present a new end-to-end deep learning framework, SSGraphCPI, for CPI prediction. The framework combines GCNN model to extract molecular topological information and BiGRU model to obtain local chemical background of SMILES/SPS. This method can extract compound/protein related information more effectively and comprehensively, which is beneficial to CPI prediction. The results show that SSGraphCPI can effectively improve the accuracy of the model and reduce the RMS error of the model on most datasets.

Furthermore, we proposed a new deep learning model SSGraphCPI2, which added protein amino acid sequence information on the basis of SSGraphCPI, and also used the BiGRU model for feature learning. The results show that the RMS error and loss value on most datasets are significantly reduced, indicating that this model can also effectively improve the accuracy of CPI prediction.

## Figures and Tables

**Table 1:** RMS errors for different models on the Test dataset.

Models	Representation of Compound	Representation of Protein	RMS Error	*R* ^2^
GRU/GCNN-CNN	molecular graph (GCNN)	SPS sequence	1.62	0.28
GCNNet	molecular graph (GCNN) + SMILES	Amino acid sequence	0.95	0.51
GATNet	molecular graph (GCNN + Attention) + SMILES	Amino acid sequence	0.91	0.57
SSGraphCPI	molecular graph (GCNN + Attention) + SMILES	SPS sequence	**0.86**	**0.63**
SSGraphCPI2	molecular graph (GCNN + Attention) + SMILES	SPS sequence + Amino acid sequence	**0.85**	**0.66**

**Table 2:** RMS errors for different models on ER dataset.

Models	Representation of Compound	Representation of Protein	RMS Error	*R* ^2^
GRU/GCNN-CNN	molecular graph (GCNN)	SPS sequence	2.33	0.036
GCNNet	molecular graph (GCNN) + SMILES	Amino acid sequence	2.29	0.048
GATNet	molecular graph (GCNN + Attention) + SMILES	Amino acid sequence	2.14	0.044
SSGraphCPI	molecular graph (GCNN + Attention) + SMILES	SPS sequence	**2.06**	**0.053**
SSGraphCPI2	molecular graph (GCNN + Attention) + SMILES	SPS sequence + Amino acid sequence	2.12	0.045

**Table 3:** RMS errors for different models on Channel dataset.

Models	Representation of Compound	Representation of Protein	RMS Error	*R* ^2^
GRU/GCNN-CNN	molecular graph (GCNN)	SPS sequence	2.62	0.019
GCNNet	molecular graph (GCNN) + SMILES	Amino acid sequence	2.17	0.045
GATNet	molecular graph (GCNN + Attention) + SMILES	Amino acid sequence	2.26	0.039
SSGraphCPI	molecular graph (GCNN + Attention) + SMILES	SPS sequence	**2.11**	**0.048**
SSGraphCPI2	molecular graph (GCNN + Attention) + SMILES	SPS sequence + Amino acid sequence	2.23	0.041

**Table 4:** RMS errors of different models on the GPCR dataset.

Models	Representation of Compound	Representation of Protein	RMS Error	*R* ^2^
GRU/GCNN-CNN	molecular graph (GCNN)	SPS sequence	2.44	0.026
GCNNet	molecular graph (GCNN) + SMILES	Amino acid sequence	2.45	0.026
GATNet	molecular graph (GCNN + Attention) + SMILES	Amino acid sequence	2.37	0.035
SSGraphCPI	molecular graph (GCNN + Attention) + SMILES	SPS sequence	**2.24**	**0.039**
SSGraphCPI2	molecular graph (GCNN + Attention) + SMILES	SPS sequence + Amino acid sequence	**2.19**	**0.042**

**Table 5:** RMS errors of different models on the Kinase dataset.

Models	Representation of Compound	Representation of Protein	RMS Error	*R* ^2^
GRU/GCNN-CNN	molecular graph (GCNN)	SPS sequence	2.98	0.011
GCNNet	molecular graph (GCNN) + SMILES	Amino acid sequence	2.76	0.014
GATNet	molecular graph (GCNN + Attention) + SMILES	Amino acid sequence	2.73	0.015
SSGraphCPI	molecular graph (GCNN + Attention) + SMILES	SPS sequence	**2.64**	**0.018**
SSGraphCPI2	molecular graph (GCNN + Attention) + SMILES	SPS sequence + Amino acid sequence	**2.47**	**0.024**

**Table 6:** The first 30 compounds known to interact with EGF proteins.

id	BindingDB_id	PubChemCID	Compound Molecular Formula
1	BDBM4343	736236	C_11_H_9_NO_3_
**2**	**BDBM4282**	**5328751**	**C_12_H_7_N_3_**
3	BDBM4279	37583	C_11_H_5_N_3_
4	BDBM4377	836	C_9_H_11_NO_4_
5	BDBM4320	746495	C_12_H_10_N_2_O_3_
6	BDBM4348	720879	C_11_H_9_NO_4_
7	BDBM4381	228618	C_10_H_8_N_4_O
8	BDBM4325	5614	C_18_H_22_N_2_O
9	BDBM4383	54212223	C_10_H_7_N_3_O
10	BDBM4012	5328588	C_38_H_38_N_4_O_2_S_2_
11	BDBM4013	5328589	C_17_H_16_N_2_OS
12	BDBM3320	5328066	C_15_H_16_N_6_
13	BDBM3972	5328551	C_22_H_20_N_2_O_4_S_2_
**14**	**BDBM4337**	**5328787**	**C_11_H_6_N_2_O**
**15**	**BDBM3990**	**5328569**	**C_34_H_30_N_4_O_2_S_2_**
16	BDBM3991	5328570	C_20_H_14_N_4_S_2_
17	BDBM4041	5328617	C_30_H_24_N_6_O_2_S_2_
18	BDBM4014	5328590	C_11_H_12_N_2_OS
19	BDBM4407	5328824	C_16_H_15_N_3_
20	BDBM3956	5328535	C_10_H_9_NO_2_S
21	BDBM3356	5328102	C_20_H_18_N_4_O_2_Se_2_
22	BDBM3971	5328550	C_20_H_16_N_2_O_4_S_2_
23	BDBM3333	5328079	C_14_H_12_N_6_O_2_
24	BDBM3258	5328015	C_16_H_15_N_3_O
25	BDBM3338	5328084	C_15_H_12_F_3_N_5_
26	BDBM3275	5328024	C_15_H_12_N_4_O_2_
27	BDBM3340	5328086	C_15_H_12_F_3_N_5_
28	BDBM4003	5328580	C_38_H_34_N_4_O_6_S_2_
29	BDBM4405	720610	C_17_H_17_N_3_
30	BDBM3964	5328543	C_12_H_13_NO_2_S

**Table 7:** Representation of the protein SPS sequence.

Secondary Structure			Solvent Exposure		Property				Length		
Alpha	Beta	Coil	Not Exposed	Exposed	Non-polar	Polar	Acidic	Basic	Short	Medium	Long
A	B	C	N	E	G	T	D	K	S	M	L

## Data Availability

Not applicable.

## References

[1] Keiser M.J., Setola V., Irwin J.J., Laggner C., Abbas A.I., Hufeisen S.J., Jensen N.H., Kuijer M.B., Matos R.C., Tran T.B. (2009). Predicting new molecular targets for known drugs. Nature.

[2] Lounkine E., Keiser M.J., Whitebread S., Mikhailov D., Hamon J., Jenkins J.L., Lavan P., Weber E., Doak A.K., Côté S. (2012). Large-scale prediction and testing of drug activity on side-effect targets. Nature.

[3] Medina-Franco J.L., Giulianotti M.A., Welmaker G.S., Houghten R.A. (2013). Shifting from the single to the multitarget paradigm in drug discovery. Drug Discov.Today.

[4] Scannell J.W., Blanckley A., Boldon H., Warrington B. (2012). Diagnosing the decline in pharmaceutical R&D efficiency. Nat. Rev. Drug Discov..

[5] Weininger D. (1988). SMILES, a chemical language and information system. 1. Introduction to methodology and encoding rules. J. Chem. Inf. Comput. Sci..

[6] You J., Liu B., Ying Z., Pande V., Leskovec J. (2018). Graph convolutional policy network for goal-directed molecular graph generation. arXiv.

[7] Bredel M., Jacoby E. (2004). Chemogenomics: An emerging strategy for rapid target and drug discovery. Nature Rev. Genet..

[8] Jacob L., Vert J.-P. (2008). Protein-ligand interaction prediction: An improved chemogenomics approach. Bioinformatics.

[9] Yamanishi Y., Araki M., Gutteridge A., Honda W., Kanehisa M. (2008). Prediction of drug–target interaction networks from the integration of chemical and genomic spaces. Bioinformatics.

[10] Bleakley K., Yamanishi Y. (2009). Supervised prediction of drug–target interactions using bipartite local models. Bioinformatics.

[11] Van Laarhoven T., Nabuurs S.B., Marchiori E. (2011). Gaussian interaction profile kernels for predicting drug–target interaction. Bioinformatics.

[12] Hattori M., Okuno Y., Goto S., Kanehisa M. (2003). Development of a chemical structure comparison method for integrated analysis of chemical and genomic information in the metabolic pathways. J. Am. Chem. Soc..

[13] Bengio Y., Courville A., Vincent P. (2013). Representation learning: A review and new perspectives. IEEE Trans. Pattern Anal. Mach. Intell..

[14] Mikolov T., Chen K., Corrado G., Dean J. (2013). Efficient estimation of word representations in vector space. arXiv.

[15] Öztürk H., Özgür A., Ozkirimli E. (2018). DeepDTA: Deep drug–target binding affinity prediction. Bioinformatics.

[16] Öztürk H., Ozkirimli E., Özgür A. (2019). WideDTA: Prediction of drug-target binding affinity. arXiv.

[17] Woźniak M., Wołos A., Modrzyk U., Górski R.L., Winkowski J., Bajczyk M., Szymkuć S., Grzybowski B.A., Eder M. (2018). Linguistic measures of chemical diversity and the “keywords” of molecular collections. Sci. Rep..

[18] Nguyen T., Le H., Quinn T.P., Nguyen T., Le T.D., Venkatesh S. (2021). GraphDTA: Predicting drug–target binding affinity with graph neural networks. Bioinformatics.

[19] Scarselli F., Gori M., Tsoi A.C., Hagenbuchner M., Monfardini G. (2008). The graph neural network model. IEEE Trans. Neural Netw..

[20] Kipf T.N., Welling M. (2016). Semi-supervised classification with graph convolutional networks. arXiv.

[21] Karimi M., Wu D., Wang Z., Shen Y. (2019). DeepAffinity: Interpretable deep learning of compound–protein affinity through unified recurrent and convolutional neural networks. Bioinformatics.

[22] LeCun Y., Bengio Y., Hinton G. (2015). Deep learning. Nature.

[23] Xiong H.Y., Alipanahi B., Lee L.J., Bretschneider H., Merico D., Yuen R.K., Hua Y., Gueroussov S., Najafabadi H.S., Hughes T.R. (2015). The human splicing code reveals new insights into the genetic determinants of disease. Science.

[24] Ma J., Sheridan R.P., Liaw A., Dahl G.E., Svetnik V. (2015). Deep neural nets as a method for quantitative structure–activity relationships. J. Chem. Inf. Model..

[25] Liu T., Lin Y., Wen X., Jorissen R.N., Gilson M.K. (2007). BindingDB: A web-accessible database of experimentally determined protein–ligand binding affinities. Nucleic Acids Res..

[26] Kuhn M., von Mering C., Campillos M., Jensen L.J., Bork P. (2007). STITCH: Interaction networks of chemicals and proteins. Nucleic Acids Res..

[27] Suzek B.E., Wang Y., Huang H., McGarvey P.B., Wu C.H., Consortium U. (2015). UniRef clusters: A comprehensive and scalable alternative for improving sequence similarity searches. Bioinformatics.

[28] Landrum G. (2013). Rdkit documentation. Release.

[29] Tian K., Shao M., Wang Y., Guan J., Zhou S. (2016). Boosting compound-protein interaction prediction by deep learning. Methods.

[30] Finn R.D., Bateman A., Clements J., Coggill P., Eberhardt R.Y., Eddy S.R., Heger A., Hetherington K., Holm L., Mistry J. (2014). Pfam: The protein families database. Nucleic Acids Res..

[31] Cheng Z., Zhou S., Wang Y., Liu H., Guan J., Chen Y.-P.P. (2016). Effectively identifying compound-protein interactions by learning from positive and unlabeled examples. IEEE/ACM Trans. Comput. Biol. Bioinform..

[32] Tabei Y., Yamanishi Y. (2013). Scalable prediction of compound-protein interactions using minwise hashing. BMC Syst. Biol..

[33] Magnan C.N., Baldi P. (2014). SSpro/ACCpro 5: Almost perfect prediction of protein secondary structure and relative solvent accessibility using profiles, machine learning and structural similarity. Bioinformatics.

[34] Sutskever I., Vinyals O., Le Q.V. (2014). Sequence to sequence learning with neural networks. Adv. Neural Inf. Process. Syst..

[35] Xu Z., Wang S., Zhu F., Huang J. Seq2seq fingerprint: An unsupervised deep molecular embedding for drug discovery. Proceedings of the 8th ACM International Conference on Bioinformatics, Computational Biology, and Health Informatics.

[36] Cho K., Van Merriënboer B., Bahdanau D., Bengio Y. (2014). On the properties of neural machine translation: Encoder-decoder approaches. arXiv.

[37] Wang Z., Chang S., Yang Y., Liu D., Huang T.S. Studying very low resolution recognition using deep networks. Proceedings of the IEEE Conference on Computer Vision and Pattern Recognition.

[38] Sutskever I., Martens J., Dahl G., Hinton G. On the importance of initialization and momentum in deep learning. Proceedings of the International Conference on Machine Learning.

[39] Tian Q., Ding M., Yang H., Yue C., Zhong Y., Du Z., Liu D., Liu J., Deng Y. (2022). Predicting drug-target affinity based on recurrent neural networks and graph convolutional neural networks. Comb. Chem. High Throughput Screen..

